# Clinical exuberance of classic Kaposi's sarcoma and response to radiotherapy*

**DOI:** 10.1590/abd1806-4841.20153877

**Published:** 2015

**Authors:** Jeniffer Muñoz Trujillo, Natália Ribeiro de Magalhães Alves, Paula Mota Medeiros, Luna Azulay-Abulafia, Maria de Fátima Guimarães Scotelaro Alves, Alexandre Carlos Gripp

**Affiliations:** 1 Universidade do Estado do Rio de Janeiro (UERJ) - Rio de Janeiro (RJ), Brazil

**Keywords:** Aged, Herpesvirus 8, human, Radiotherapy, Sarcoma, Kaposi

## Abstract

Kaposi's sarcoma (KS) is a multicentric vascular neoplasm, with cutaneous and extracutaneous involvement. Different clinical and epidemiological variants have been identified. The classic form is manifested mainly in elderly men with indolent and long-term evolution, with lesions localized primarily in the lower extremities. We present two cases of classic Kaposi's sarcoma (CKS) in two female patients with extensive, exuberant skin involvement and rapid evolution, with good response to radiotherapy.

## INTRODUCTION

KS is a vascular endothelium multifocal angioproliferative disorder, of preferred mucocutaneous location, with potential to reach internal organs. It was described by Moritz Kaposi as idiopathic multiple pigmented sarcoma of the skin, in 1872.^1,2^

Currently, four clinical-epidemiological forms are recognized: classic, endemic, iatrogenic and epidemic, each one with its own natural history, preferred location and prognosis.^1,3^ CKS mainly affects the skin of lower limbs in aged people. It has a long-term course with typical clinical presentation of macules, papules, erythematous-violaceous plaques or nodules.^4^ In the absence of treatment, clinical course of KS varies from innocuous lesions seen in its classic variant to rapidly progressing and fatal lesions in epidemic KC.^1,5^

Two cases of CKS in female patients are presented, with extensive and exuberant cutaneous involvement, of rapid progression and good response to radiotherapy.

## CASE REPORT

### Case 1

**Figure 1:**
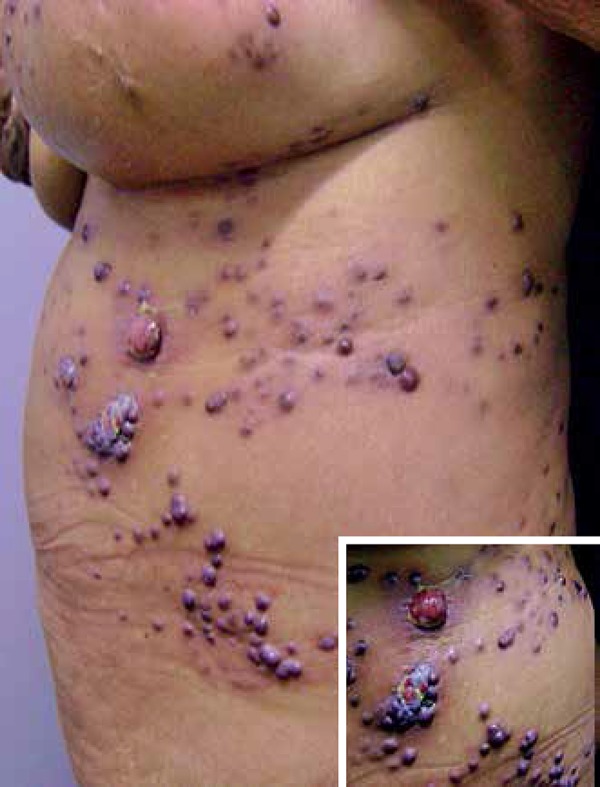
Multiple erythematous-violaceous, exophytic nodules of different diameters, located in the inframammary region and left flank. In greater detail, ulceration on the surface of one the nodules

Female patient, 82 years old, black, born in and from Rio de Janeiro. She has presented for 9 months painful and pruritic nodular lesions, initially in the left inframammary region which spread to abdomen, back and buttocks regions. At the dermatological examination, she presented multiple exophytic, erythematous-violaceous nodules and tumors lesions, some ulcerated, located in the left inframammary region and left breast, left flank, back and buttocks region (Figure 1). She did not present lesions in the mucosae and internal organs.

### Case 2

**Figure 2:**
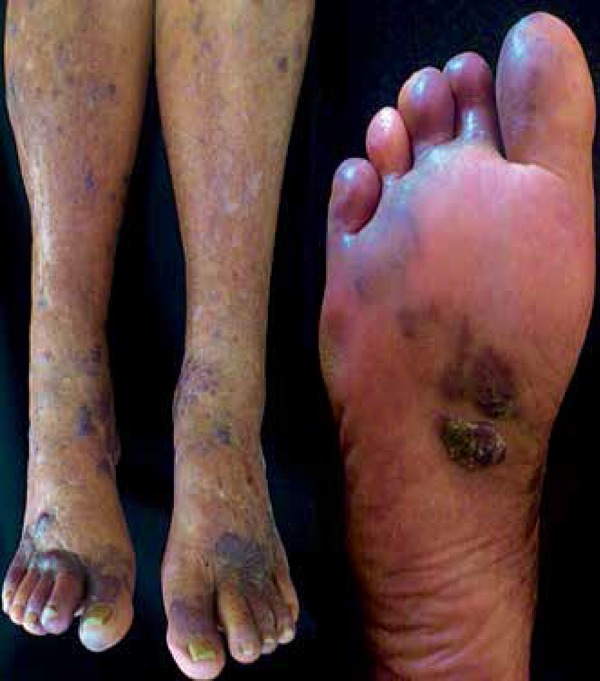
Irregular violaceous plaques involving legs and dorsum of feet. Violaceous plaques of keratotic surface, located in right plantar region

Female patient, white, 90 years old, born in and from Rio de Janeiro. She resported a condition of approximately 18 months of progression since the onset of a few violaceous nodules in the upper right limb. After one month, there was the onset of multiple lesions of similar characteristics to previous ones on the abdomen and lower and upper limbs. Edema and sensation of lower limb fatigue were associated with clinical features. At the dermatological examination, she presented plaques and violaceous nodules of different diameters located on the upper and lower limbs, trunk, dorsum of feet and plantar region (Figure 2). This last location included reports of pain.

**Figure 3:**
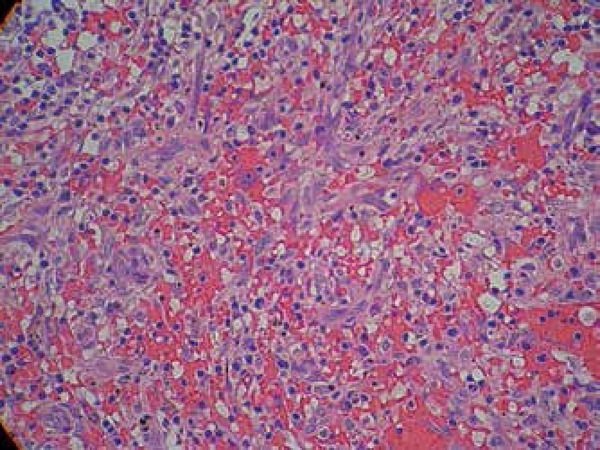
Non-encapsulated dermal nodular proliferation composed by irregular vascular channels with extravasated red blood cells and proliferation of fusiform cellsrface, located in right plantar region

In both cases a cutaneous biopsy was performed, which revealed numerous vessels of varied sizes in the dermis, exhibiting endothelial cells with nuclear hyperchromatism and some atypias, proliferation of fusiform cells, showing endothelial markers and diapedesis of red blood cells and histopathological picture compatible with Kaposi's sarcoma (Figure 3). Immunohistochemistry for HHV 8 was positive in both cases. Serologies for HIV 1 and 2, hepatitis B and C were negative. Thorax and abdomen tomography and upper digestive endoscopy did not reveal changes.

**Figure 4:**
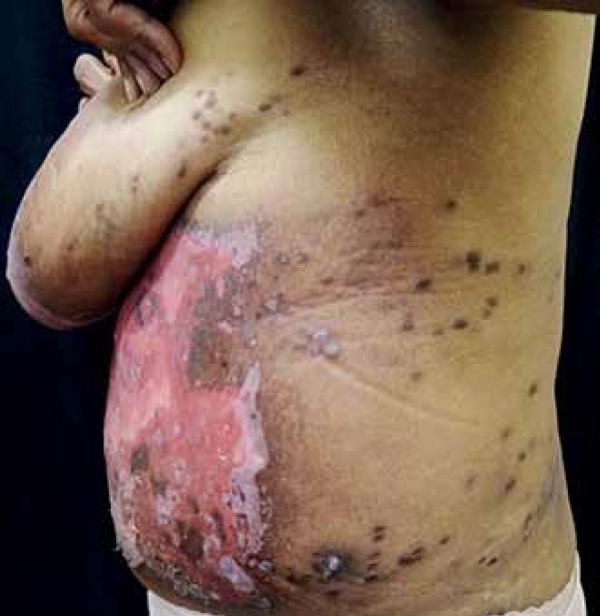
Extensive erosion due to acute radiodermititis. Hyperchromic macules and plaques can be observed in the area treated with radiotherapy (Patient 1)

Case 1 patient had the left mammary region and abdomen treated with radiotherapy, which was also used to treat the lower limbs of case 2 patient. They received three 800 centigray fractions on days 0-7-21. The first patient responded well with reduction of some lesions and regression of others, but had radiodermititis in the treated site (Figure 4). Two months after radiotherapy, new nodular lesions appeared in these sites.

**Figure 5:**
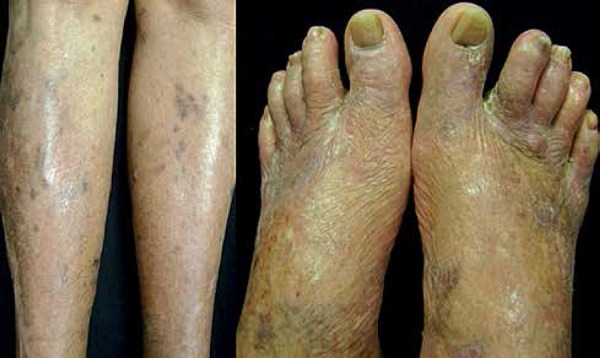
Resolution of KS lesions, with only hyperchromic macules remaining after three sessions of radiotherapy (Patient 2)

In case 2, there was satisfactory response to radiotherapy, with improvement of lesions and symptomatology (Figure 5).

## DISCUSSION

Kaposi's sarcoma is a low-grade vascular tumor that may involve the skin, mucosa and viscera, developing in one of four different clinical-epidemiological contexts.^6^ The patients described presented CKS in accordance with clinical history, age bracket and exclusively cutaneous location.

Chang et al. discovered the human herpesvirus 8 (HHV8) in 93% of KS lesions through Polymerase Chain Reaction in 1994.^6,7^ However, its role is not clear, whether a causative agent or present only in those lesions with a function not yet defined,.^4^ In our patients, HHV8 was detected as described in literature.

CKS is a rare disease with indolent course, involving preferably men between 40 and 70 years of age and of Mediterranean and Eastern Europe origin.^6^ It involves mainly the skin, starting with violaceous macules in the distal portion of lower limbs, progressing to plaques, nodules and tumors lesions.^4^ As the disease develops, lesions become hardened, with irregular surface, where there may be ulceration and perilesional edema.^4,6^ Our patients are an exception to this pattern regarding sex, geographic origin and distribution of lesions.

About 10% of patients suffer mucous and visceral involvement, usually compromising lymph nodes and the gastrointestinal tract.^8^

The course of CKS is slow, but progression of lesions may be variable; macules or tumors may not change for months to years or present a fast growth in weeks and disseminate. A rapid progression may lead to central necrosis and ulceration of lesions, as in case 2.^9^

Unlike epidemic KS, CKS does not have a universally accepted staging classification. The staging system proposed by Brambilla et al in 2003 is based on objective criteria which follow closely the clinical variability of CKS. This system is composed of four stages based on cutaneous lesions: location, presence or absence of complications and visceral involvement (Chart 1).^9^ Both cases 1 and 2 were evaluated as stage IVB due to fast progression, characteristics, location and behavior of lesions, presenting local complications nevertheless, Case 1 with pain and ulceration and Case 2 with pain in the plantar region upon walking.

**Chart 1:** Staging of Classic Kaposi's Sarcoma

Stage	Cutaneous Lesions	Location	Behavior	Progression	Complications
I. Maculonodular	Macules or nodules or both	Lower limbs	Non-aggressive	Slow (A) Fast (B)	aLymphedema, hemorrhage, pain, functional involvement, ulceration
II. Infiltrative	Plaques	Lower limbs	Locally aggressive	Slow (A) Fast (B)	
III. Florid	Angiomatous nodules and plaques	Extremities, particularly the lower ones	Locally aggressive	Slow (A) Fast (B)	
IV. Disseminated	Angiomatous nodules and plaques	Extremities, trunk, head	Disseminated, aggressive	Fast (B)	

Stage V: visceral involvement (oropharynx, gastrointestinal tract, lymph nodes, bone marrow, lungs).

Fast: Increase in total number of nodules/plaques or in total area of plaques, in 3 months.

a All prevail in stage III and IV; lymphedema and lymphorrhea are observed in stage II and rarely in stage I

**Source:** Bambrilla et al 2003^9^

In localized forms of CKS, the available therapeutic alternatives are radiotherapy, surgery, intralesional injections and observation. When there is internal involvement or local complications, systemic therapy with antiproliferative medication is required.^10^ Even though there is not enough evidence in literature for recommending a therapeutic strategy, radiotherapy studies have reported a complete response in approximately 60-93% of the cases, being effective in localized cutaneous forms.^10^ Although the disease of the patients here presented was in stage IV, radiotherapy was the option, justified by the age of the patients.

In both cases clinical exuberance and rate of progression emphasized the idea of CKS variability. Over recent years an increase in reported cases of CKS with different behavior has been noted. All data about these cases must be gathered for evaluating possible new clinical KS classifications.
